# Association of smoking with restenosis and major adverse cardiac events after coronary stenting: A meta-analysis

**DOI:** 10.12669/pjms.314.7495

**Published:** 2015

**Authors:** Rui-ting Hu, Jie Liu, You Zhou, Bang-li Hu

**Affiliations:** 1 Rui-ting Hu, MD. Minzu Affiliated Hospital of Guangxi Medical University, 530001 Nanning, China; 2 Jie Liu, MD, PhD. Department of Cardiology, People’s Hospital of Guangxi Zhuang Autonomous Region, 530021 Nanning, China; 3 You Zhou, MD, PhD. Minerva Foundation Institute for Medical Research; FI-00290 Helsinki, Finland; 4 Bang-li Hu, MD. First Affiliated Hospital of Guangxi Medical University, 530021 Nanning, China

**Keywords:** Smoking, In stent restenosis, Major adverse cardiac events, Major adverse cardiac and cerebrovascular events, Meta-analysis

## Abstract

**Background and Objective:**

The association between smoking and clinical outcomes after coronary stenting is controversial. The aim of this meta-analysis was to assess the association between smoking and in stent restenosis (ISR), major adverse cardiac events (MACE), or major adverse cardiac and cerebrovascular events (MACCE) after coronary stenting.

**Methods:**

A search for studies published before December 2014 was conducted in PubMed, Embase, and Cochrane library. An inverse random weighted meta-analysis was conducted using logarithm of the odds ratio (OR) and its standard error for each study.

**Results:**

Ten studies investigated the association between smoking and ISR. Overall, smoking was not associated with ISR (OR: 1.05, 95% CI: 0.79–1.41; I^2^ = 47.8%). Subgroup analysis also failed to show a significant association between smoking and ISR risk regardless of bare metal stent (BMS) and drug-eluting stent (DES) implantation. Eight studies explored the association between smoking and MACE, but no association was found (OR: 0.92, 95% CI: 0.77–1.10; I^2^ = 25.5%), and subgroup analysis revealed that no distinct difference was found between BMS and DES implantation. Three studies investigated the association between smoking and MACCE and significant association was found (OR: 2.09, 95% CI: 1.43–3.06; I^2^ = 21.6%).

**Conclusions:**

Our results suggest that in patients undergoing percutaneous coronary intervention with stent implantation, smoking is not associated with ISR and MACE; however, smoking is an independent risk factor for MACCE.

## INTRODUCTION

Findings of previous studies strongly suggest that cigarette smoking is a preventable risk factor for coronary artery disease (CAD) and is strongly associated with cardiovascular-related morbidity and mortality. Previous study showed the negative effects of smoking on late mortality in patients who underwent coronary artery bypass grafting.^1^ However, conflicting results were obtained in patients who underwent percutaneous coronary intervention (PCI). Several studies showed that cigarette smoking was associated with a lower rate of subsequent target lesion revascularization (TLR).^2^ In addition, among patients with acute myocardial infarction (MI), smokers have an even better short-term survival.^3^ Furthermore, smoking appears to enhance the antiplatelet effect of clopidogrel.^4^

PCI with stent implantation, such as bare metal stent (BMS) or drug-eluting stent (DES) implantation, has been widely used to treat stenotic coronary arteries found in coronary heart disease. However, there are also paradoxical results about the effect of smoking on clinical outcomes after PCI, such as in stent restenosis (ISR),^5^,^6^ major adverse cardiac events (MACE), and major adverse cardiac and cerebrovascular events (MACCE).^7^,^8^ The inconsistent results were generally caused by factors such as different baseline characteristics of patients and small sample size; therefore, to determine the impact of smoking on ISR and MACE after coronary stenting, we conducted a meta-analysis by incorporating adjustments for relevant confounding factors.

## METHODS

### Search strategy

In order to find all the studies that examined the association between smoking and ISR and MACE after stent implantation, we systematically searched the Cochrane clinical trials database, Medline (PubMed), Embase, and Google scholar for studies published before December 2014. We used the following search terms: “smoking” or “cigarette”, “in stent restenosis” or “ISR”, “major adverse cardiac events” or “MACE”, and “coronary”. The search was not limited by language or publication status. We searched the references of all retrieved publications again to trace additional relevant studies. Moreover, the relevant review articles and their references were checked as well. In cases of multiple publications of the same or overlapping cohort, only the studies with the largest sample size were included. Potentially relevant articles were then screened by at least two independent reviewers; disagreements were resolved by discussion or upon consensus from the third reviewer.

### Inclusion and exclusion criteria

The identified studies met the following criteria: (1) The study design was an observational study in human beings; (2) the study investigated the association between smoking and ISR and MACE after stent implantation; (3) the study provided data about the effect of smoking on ISR or MACE (the odds ratio [OR] and 95% confidence interval [95% CI]) from multivariate analysis; (4) ISR was defined as ≥50% diameter stenosis of the culprit lesion by quantitative coronary analysis; and (5) the duration of follow-up was at least 6 months. MACE varied slightly in the various studies, but generally, it consisted of cardiac death, myocardial infarction, and repeat revascularization; MACCE was MACE but included stroke or cerebrovascular accidents. Exclusion criteria were as follows: laboratory studies, review articles, animal studies, and studies with a follow-up period shorter than 6 months.

### Data extraction and quality assessment

Two blinded reviewers independently performed data extraction. Disagreements between the reviewers were resolved through discussion or by the third reviewer. The extracted data included: (1) the first author’s last name, publication year, and origin of the studied population; (2) characteristics of the study population, stent types, and duration of follow-up; (4) study design; and (5) adjustments for confounding factors. We only choose data on current smoking when studies provided both former smoking and current smoking data. The quality of included studies was assessed by the Systematic Appraisal of Quality for Observational Research (SAQOR) criteria.^9^ The instrument recorded 5 criteria: (1) the sample is representative of the population from which it was drawn; (2) the source of the sample is clearly stated; (3) the sampling method is described; (4) the sample size is appropriate to determine statistical significance for primary outcomes; and (5) the inclusion and exclusion criteria are stated and justified.

### Statistical analysis

Software STATA version 11.0 (Stata Corporation, College Station, TX, USA) was used for all analysis. Data are expressed as OR and 95% CI. The individual estimates of the log OR with its standard error for each study were combined to obtain the summary estimate of the OR of ISR and MACE by using the inverse variance weighted method. We assessed the heterogeneity between studies in this meta-analysis by the Cochran Q test. We also calculated the inconsistency index I^2^ to quantify heterogeneity. I^2^ was documented for the percentage of the observed variation between studies which was caused by heterogeneity rather than chance. In addition, to explore sources of heterogeneity, we performed a sensitivity test. Sensitivity analysis was performed to assess robustness and examine the results for possible bias. Subgroup analysis was carried out to assess more narrowly drawn subsets of the studies. To investigate whether publication bias might affect the validity of the estimates, funnel plots were constructed. Funnel plot asymmetry was assessed by Egger’s linear regression test. *P* values < 0.05 indicated statistical significance.

## RESULTS

### Literature search

**Fig. 1:**
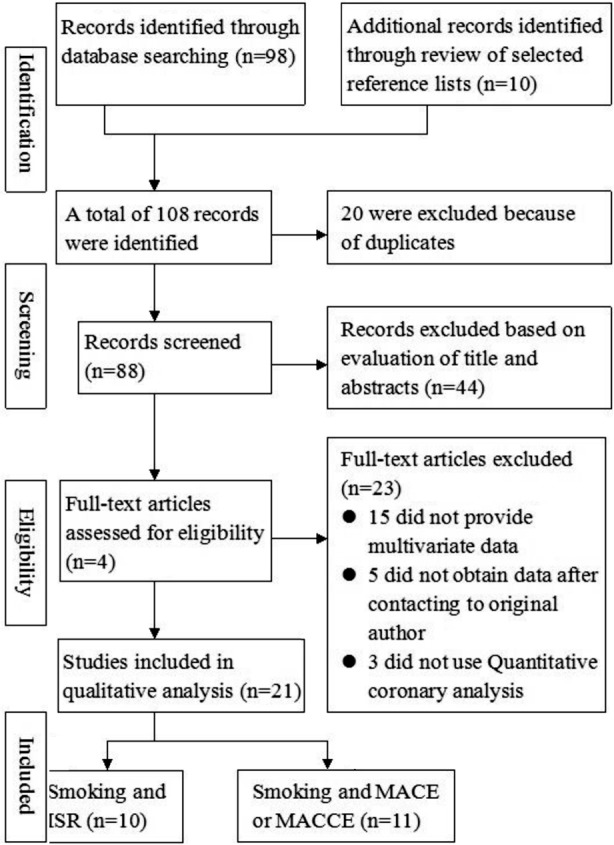
Flow chart of study selection based on the inclusion and exclusion criteria.

The primary literature search retrieved 108 records. After title or abstract screening and the full text evaluation, 21 studies were finally selected. Among them, 12 studies had a prospective design, 8 studies had a retrospective design, and one study was a randomized controlled trial. A flow diagram of the selection process is shown in Fig.1.

### Study characteristics and quality assessment

Ten studies including a total of 3484 patients investigated the association between smoking and ISR.^5^,^6^,^10^-^17^ Eight studies including a total of 5406 patients investigated the association between smoking and MACE.^7^,^18^-^24^ Three studies^8^,^25^,^26^ including a total of 2581 patients used MACCE as endpoints. Additional information of patients’ demographics is listed in Table-I. The quality assessment showed that all the included studies met the SAQOR criteria.

**Table-I:** Characteristics of included studies of ISR.

Study	Year	Design	Stent	Age	Male	Total	FU	Confounding factors
Niroomand F, et al. (2004)		Retrospective	BMS	63.5	225(100.0)	225	6m	Age, BMI, Diabetes, Hypertension, Previous MI, Previous CABG, Previous PTCA, Number of diseased vessels, Impaired LV function,
Rittersma SZ, et al. (2004)		Prospective	BMS	58	278(80.6)	345	6-10m	Hypertension, Unstable angina, Diabetes mellitus, Chronic total occlusion, Stent length, Statin, Reference diameter, MLD, CRP
Choi EY, et al. (2005)		Prospective	BMS	60.3	148(72.9)	203	6m	Age, Female, Hypertension, Smoking BMI, HbA1c, Hypertriglyceridemia, ACS, Multivessel Disease, Post MLD, AGE, Long Stent
Kamitani T, et al. (2005)		Prospective	BMS	61.8	97(89.0)	109	6m	Age, Gender, Hypertension, DM BMI, Hyperlipidemia, Lp(a) Reference diameter, Lesion length
Hong SJ, et al. (2006)		Retrospective	DES	62.2	70(33.2)	211	6m	Age, Women, BMI, Unstable angina, Stable angina, Left ventricular ejection fraction, Hypertension, Hypercholesterolaemia, Lesion location, Quantitative coronary angiography, Laboratory analysis
Hong SN, et al. (2007)		Prospective	B/D	61	178(73)	245	6m	Age, Hypertension, Diabetes, Smoking Dyslipidemia, CRP, Fibrinogen, DES, NT–pro-BNP
Kim JS, et al. (2009)		Retrospective	DES	56	394(70.7)	557	9m	Age, Male, Hypertension, DM, Hyperlipidemia, ACS Multivessel disease B2 or C lesion, Stent diameter, Stent length, Stent fracture
Li B, et al. (2011)		Prospective	DES	60.4	120(58.3)	210	6m	Age, Male, BMI, Diabetes, Hypertension, Hypercholesterolemia, Statins, Reference diameter, MLD, Stent diameter, Length of stent segment,
Xu YL, et al. (2011)		Prospective	DES	57	237(78.2)	303	8m	Age, Gender, Hypertension, DM, BMI, Previous MI, Multivessel disease, Multiple complex lesion, Target lesion stenosis, Target lesion length,
Kuwano T, et al. (2011)		Retrospective	B/D	67	859(79.8)	1076	8m	Age, Gender, Hypertension, DM, BMI, Hyperlipidemia, Renal insufficiency, Stent length, Statin, Reference diameter, MLD, DES
Fujiwara K, et al. (2002)		Retrospective	BMS	64.2	268(83.0)	323	6m	DM, Age, Female, Hypertension, Hyperlipidemia, Prior infarction Anterior MI,TIMI, Cardiogenic shock, Multiple vessel disease
Kralev S, et al. (2009)		Prospective	BMS	65	291(73.9)	394	6m	DM, Usage of GP IIb/IIIa, HLP, Hypertension, Obesity, Family history, Male, Age, CK, TNI
Hong SJ, et al. (2010)		RCT	DES	65.9	125(74.0)	169	3y	Women, Stable angina, Unstable angina, Hypertension, Hypercholesterolemi, PES implantation, Insulin treatment, Stent length, Stent diameter, Post-PCI RD, Post-PCI MLD, LVEF
Gurvitch R, et al. (2010)		Prospective	DES	62.6	404(71.6)	564	12m	DES, Propensity score, Age, Female, Diabetes, Hypertension, Renal failure, Cerebrovascular disease, Cardiogenic shock
Nakamura M, et al. (2010)		Prospective	DES	66.2	641(72.1)	889	3y	Hemodialysis,diabetes,Ostial,Multi-vessel disease, Ejection fraction, Hyperlipidemia
Shimony A, et al. (2010)		Retrospective	DES	65	1033(73.9)	1397	1.8y	Lower SI DES,DM, Hypertension, Clopidrogel use, Age, Multivessel disease, gender, Dyslipidemia, LAD
Matsumoto I, et al. (2011)		Prospective	B/D	67.7	537(78.2)	687	6m	Age, Male, BMI,L/Hratio, Triglyceride, HbA1c, CRP, GFR, Statin, DES
Ogita M, et al. (2011)		Retrospective	BMS	64.7	628(63.9)	983	6m	ISR, Age, Multivessel disease, Diabetes, Prior MI, Prior PCI, LVEF, HbA1c, eGFR, Insulinusage
Hung WC, et al. (2010)		Prospective	B/D	64.6	147 (76.2)	193	15.3m	Age, Gender, DM, BMI, Hypertension, Fasting glucose, HbA1C, Cholesterol, Creatinine, Plasma adiponectin, CRP
Sherif MA, et al. (2011)		Prospective	DES	57.4	1493(68.8)	2174	12.5m	Age, Gender, Hypertension, DM, BMI, Hyperlipidemia, Renal insufficiency, Stent length, Statin, Reference diameter, MLD,STEMI, Heart failure
Meliga E, et al. (2012)		Retrospective	B/D	36.3	189(88.3)	214	12m	Gender, Family history, Hypertension, Hypercholesterolemia, Diabetes, STEMI, Left ventricular ejection fraction, Multivessel disease

BMS, bare metal stent; DES, drug eluting stent; B/D, BMS and DES; FU, follow-up.

### ISR

**Fig. 2:**
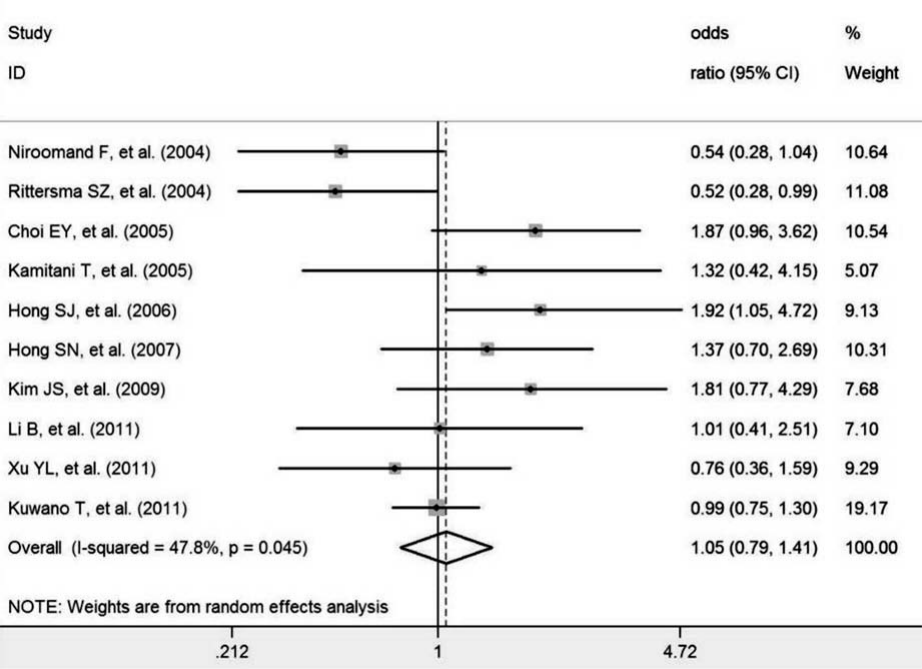
Meta-analysis of the association between smoking and ISR.

Overall, smoking was not associated with ISR after coronary stenting (OR: 1.05, 95% CI: 0.79–1.41) (Fig.2). There was moderate heterogeneity across the studies (I^2^ = 47.8%, *P* = 0.045). No publication bias was detected (Egger’s test: *P* = 0.607).

We performed a sensitivity analysis to address the relative importance of each study. After exclusion of each study in turn, no significant association was found between smoking levels and ISR, in agreement with the overall analysis. Subgroup analysis showed no significant association between smoking and ISR risk after BMS implantation (OR: 0.88, 95% CI: 0.45–1.73) and DES implantation (OR: 1.15, 95% CI: 0.65–2.03).

### MACE

**Fig. 3:**
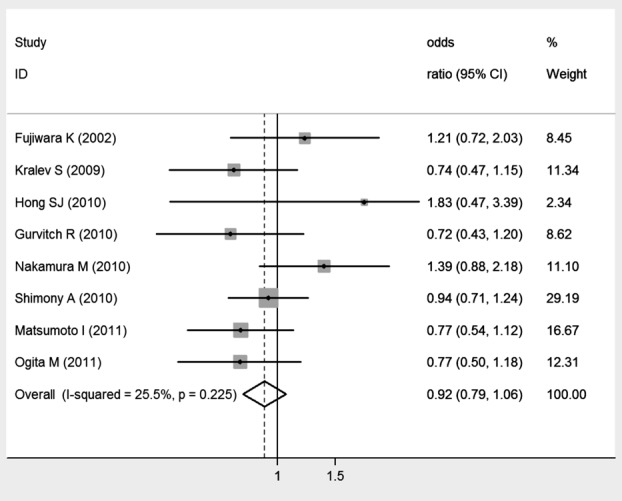
Meta-analysis of the association between smoking and MACE.

Overall, smoking was not associated with MACE after coronary stenting (OR: 0.92, 95% Fig.3. No publication bias was detected (Egger’s test: *P* = 0.114).

Subgroup analysis also failed to detect a significant association between smoking and MACE risk after BMS implantation (OR: 0.86, 95% CI: 0.64–1.15) and DES implantation (OR: 1.16, 95% CI: 0.82–1.63). Similar subgroup analysis results were found regarding different follow-up lengths (less than 1 year, OR: 0.83, 95% CI: 0.67–1.03; more than 1 year, OR: 1.14, 95% CI: 0.76–1.42).

Sensitive analysis by removing Shimony et al. data, the results were similar to the main results (OR: 0.91, 95% CI: 0.76–1.08) and without significant heterogeneity (I^2^ = 35.8%, *P* = 0.155), suggesting the robust of the results.

### MACCE

**Fig. 4:**
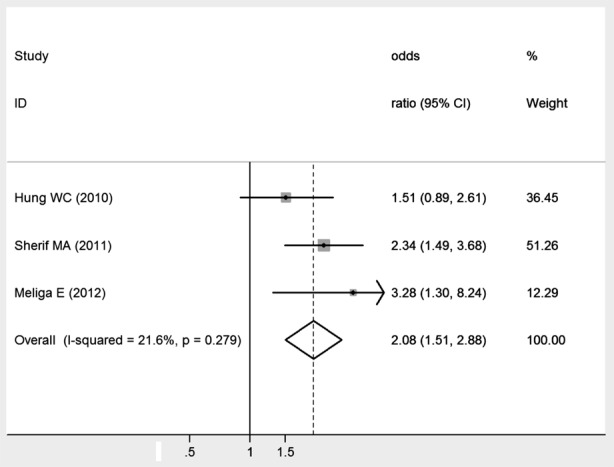
Meta-analysis of the association between smoking and MACCE.

There was a significant association between smoking and MACCE after coronary stenting (OR: 2.08, 95% CI: 1.51–2.88), and no significant heterogeneity between the studies (I^2^ = 21.6%, *P* = 0.279). Fig.4. Egger’s test suggested little publication bias (*P* = 0.721).

## DISCUSSION

### Smoking and cardiovascular disease

A great number of cardiovascular diseases are associated with smoking. Research has confirmed that smoking damages the blood vessels and influences all phases of atherosclerosis, from endothelial dysfunction to acute clinical events.^27^ The exact toxic components of cigarette smoke and the mechanisms involved in smoking-related cardiovascular dysfunction are largely unknown; however, smoking increases inflammation, thrombosis, and oxidation of low-density lipoprotein cholesterol.^28^ Experimental and clinical data also showed that cigarette smoke exposure increases oxidative stress as a potential mechanism for initiating cardiovascular dysfunction.^29^,^30^

### Smoking and ISR

Generally, cigarette smoking is considered to be associated with CAD progression and restenosis following angioplasty because of its effects on endothelial and platelet function. As a result, patients are routinely advised to cease smoking before coronary angioplasty. However, published studies have reported conflicting results. Hong et al.^5^ studied 840 patients with DES implantation and reported that current smoking was a predictor of restenosis in diabetic patients (OR: 1.923, 95% CI: 1.055– 4.725). Ma et al.^31^ also reported that current smoking increases the risk of restenosis in ST-segment elevation MI patients undergoing sirolimus-eluting stent implantation. In addition, similar results were reported in patients with carotid restenosis^32^ after stent implantation.

Kuwano et al.^17^ reviewed 1076 patients who underwent coronary stenting, with a follow-up of 236 days; however, no significant association was found between current smoking and ISR. In addition, Mohan and Dhall et al.^33^ compared the restenosis rates between BMS and DES; the follow-up period was 6 to 9 months, but no significant difference in restenosis rate was found between these 2 types of stents (*P* = 0.27). Furthermore, Rittersma et al.^6^ even showed that smoking can reduce the risk of ISR (OR: 0.52, 95% CI: 0.28–0.99). The explanation for this dissociation between smoking and angiographic restenosis is that smokers have a reduced sensitivity to restenosis, and smokers are more reluctant to seek medical attention despite recurrent angina.^34^ In the present study, our results also failed to show that smoking was associated with ISR risk, regardless of BMS or DES implantation, which was similar to some previous reports.

### Smoking and MACE or MACCE

Regarding the effect of smoking on MACE, the associations were also controversial. Ogita et al.^24^ analyzed the data of 983 CAD patients with BMS implantation, and found that smoking did not increase the risk of MACE. Similar results were reported by Nakamura et al.^7^; they failed to show that smoking was an independent risk factor of MACE in patients with DES implantation. However, Meliga et al.^25^ performed a retrospective study of patients with both BMS and DES implantation with a follow-up of over 2 years and found a significant association between smoking and MACE. In a prospective study of patients undergoing DES implantation with a follow up of 12.5 months, the strongest independent predictor for MACE was smoking.^8^

In the present study, the overall results did not support the association between smoking and MACE. However, there was an association between smoking and MACCE. The definition of MACCE included stroke or cerebrovascular accidents, and the follow up length of the 3 studies with MACCE as an endpoint was longer than one year; we suppose that these may be the reasons for the different results on the association between smoking and MACE or MACCE. However, other reasons may also exist, such as smoking status, degree of smoking, or the lifestyle of patients. Furthermore, although no significant heterogeneity was found, only 3 studies investigated the association between smoking and MACCE. A reliable estimate of the association between smoking and MACCE still needs to be further investigated by a large prospective design study, considering the smoking status and other possible confounders.

Previously, Sherif et al.^8^ found that smokers may be more prone to the development of unstable plaques, and their increased risk of acute MI persists even after DES implantation. Epidemiologic studies also indicated that cigarette smoking increases the risk of acute MI and sudden cardiac death, much more than it increases the risk of angina pectoris.^35^ In contrast to the above studies, our subgroup analysis showed no significant association between smoking and MACE regardless of BMS or DES implantation, and the duration of follow-up (greater than or less than one year), which suggested that smoking did not significantly affect the incidence of death, TLR, or MI after stent implantation. We postulated at least 2 reasons to explain these differences. First, among patients with acute MI, smokers have better short-term survival. Second, smoking has been associated with a lower rate of TLR in patients undergoing PCI, and the overall incidence of MACE was subsequently reduced in smokers.^2^

To our knowledge, this is the first meta-analysis to explore the association between smoking and ISR, MACE, and MACCE after coronary stenting. Compared to previous studies, our study included more subjects, and the OR value from each study was adjusted by the relevant confounding factors, which guaranteed the robustness of results. However, some limitations need to be noted. First, the definition of smoking was inconsistent. Although we only selected current smoking patients, several studies did not specify the smoking status of the study subjects, potentially influencing the interpretation of the impact of smoking on ISR and MACE. Second, the type of DESs in the present study included sirolimus-eluting stents (SESs) and paclitaxel-eluting stents (PESs), and SESs are superior to PESs in reducing the incidences of restenosis and TLR. However, in the present study, we did not analyze their effect separately; therefore, the potential influence of different stents could not be detected. Third, several of the included studies had a retrospective design. Although our results showed that the summary results of retrospective design studies were similar to those of prospective design, the bias of retrospective design studies, such as selection bias and recall bias, should not be neglected. Fourth, although we did not find that smoking was associated with ISR or MACE risk, this null association may have been caused by the limited number of included studies for each endpoint. If more studies were included, the association could be significant. Fifth, although we used adjusted OR values to reduce the bias caused from the varied baseline clinical characteristics of patients, however, because there is a heterogeneity on post-PCI anticoagulation or pharmacological risk modification in different period in history, the bias caused by this heterogeneitymay undermine the robust of our results. Six, because the ISR, MACE and MACCE is often occur after 6 month post-PCI,^36^ we therefore only chose patients with the follow-up period over 6 months. However, the follow-up period of some included studies in much longer than 6 month, which may influence the occur of ISR and thus causes to bias. Therefore, due to the above limitations, our results should be interpreted cautiously.

In conclusion, our results suggest that in patients undergoing PCI with stent impanation, smoking is not associated with ISR and MACE; however, smoking is an independent risk factor for MACCE.
